# Testing the Empathy Theory of Dreaming: The Relationships Between Dream Sharing and Trait and State Empathy

**DOI:** 10.3389/fpsyg.2019.01351

**Published:** 2019-06-20

**Authors:** Mark Blagrove, Sioned Hale, Julia Lockheart, Michelle Carr, Alex Jones, Katja Valli

**Affiliations:** ^1^Department of Psychology, Swansea University, Swansea, United Kingdom; ^2^Swansea College of Art, University of Wales Trinity Saint David, Swansea, United Kingdom; ^3^Goldsmiths, University of London, London, United Kingdom; ^4^Department of Psychology, University of Turku, Turku, Finland; ^5^Department of Cognitive Neuroscience and Philosophy, The University of Skövde, Skövde, Sweden

**Keywords:** dreaming, empathy, social simulation, dream sharing, human bonding, human evolution and behavior, human consciousness, consciousness

## Abstract

In general, dreams are a novel but realistic simulation of waking social life, with a mixture of characters, motivations, scenarios, and positive and negative emotions. We propose that the sharing of dreams has an empathic effect on the dreamer and on significant others who hear and engage with the telling of the dream. Study 1 tests three correlations that are predicted by the theory of dream sharing and empathy: that trait empathy will be correlated with frequency of telling dreams to others, with frequency of listening to others’ dreams, and with trait attitude toward dreams (ATD) (for which higher scores indicate positive attitude). 160 participants completed online the Toronto Empathy Questionnaire and the Mannheim Dream Questionnaire. Pearson partial correlations were conducted, with age and sex partialled out. Trait empathy was found to be significantly associated with the frequency of listening to the dreams of others, frequency of telling one’s own dreams to others, and attitude toward dreams. Study 2 tests the effects of discussing dreams on state empathy, using an adapted version of the Shen (2010) state empathy scale, for 27 pairs of dream sharers and discussers. Dream discussion followed the stages of the Ullman (1996) dream appreciation technique. State empathy of the dream discusser toward the dream sharer was found to increase significantly as a result of the dream discussion, with a medium effect size, whereas the dream sharer had a small decrease in empathy toward the discusser. A proposed mechanism for these associations and effects is taken from the robust findings in the literature that engagement with literary fiction can induce empathy toward others. We suggest that the dream acts as a piece of fiction that can be explored by the dreamer together with other people, and can thus induce empathy about the life circumstances of the dreamer. We discuss the speculation that the story-like characteristics of adult human dreams may have been selected for in human evolution, including in sexual selection, as part of the selection for emotional intelligence, empathy, and social bonding.

## Introduction

In general, dreams are a novel but realistic simulation of waking social life, with a mixture of characters, motivations, scenarios, and positive and negative emotions. We propose that the sharing of dreams has an empathic effect on the dreamer and on significant others who hear and engage with the telling of the dream. This suggested post-sleep effect of dreams can be contrasted with theories of within-sleep functions, such as that dreams reflect memory processing during sleep (Walker and Van der Helm, 2009; Blagrove et al., 2011a, b; Wamsley and Stickgold, 2011, 2018; Wamsley, 2014; van Rijn et al., 2015; Eichenlaub et al., 2018; Scarpelli et al., 2019), or reflect pre-sleep emotional waking life (Schredl, 2006; Malinowski and Horton, 2014; Blagrove et al., 2019).

### Dreaming and Personal Insight

There has been much work on the effects on the dreamer of telling and discussing dreams. Edwards et al. (2013) showed that the dreamer obtains deepened self-perception and personal gains from participation in sessions that follow the Ullman (1996) group dream discussion procedure. Edwards et al. (2013) distinguished between insight about the memory sources of an item of dream content and insight about one’s waking life as a result of considering the dream. These two forms of insight, and level of engagement in discussing, exploring and working with the dream, contribute to the score on the exploration-insight subscale of the Gains from Dream Interpretation questionnaire (Heaton et al., 1998). Scores on this exploration-insight subscale were found to be very high after dream discussion and comparable to scores from Hill’s well-established therapist-led dream interpretation method (Heaton et al., 1998). Edwards et al. (2015) showed that exploration-insight scores are greater for considering dreams than for considering a recent personally significant event, where both sets of reports were discussed and explored using the Ullman procedure. Furthermore, in Blagrove et al. (2019), participants rated discussions of dreams significantly higher than discussions of daydreams on exploration-insight, and also rated the statement “I learned more about issues in my waking life” more highly from discussing a dream than from discussing a daydream. These latter results were obtained even though participants did not select the dreams, as these were collected in the sleep laboratory, whereas home dreams can often be selected for sharing on the basis of appearing to be interesting, intriguing, useful or impactful.

In Blagrove et al. (2019), after approximately 50% of Rapid Eye Movement (REM) and non-REM (NREM) dream discussions, participants were able to describe some insight about their life that had resulted from the discussion. These insights were often not astounding, but showed that the dream content could act as a reminder, a reference to what might be being ignored in waking life. Many of the references to waking life were metaphorical, which accords with the extensive literature on dreams and metaphor. For example, Davidson and Lynch (2012) provide experimental evidence for the figurative or metaphorical expression of waking life emotional experiences in dream content, as well as literal representations, and Malinowski and Horton (2015) detail how metaphor and hyperassociativity are imagination mechanisms behind emotion and memory assimilation in sleep and dreaming. On this, using the theory of conceptual metaphor, Lakoff (1993) shows how metaphors structure cognition in waking life and in dreaming, in that abstract ideas are thought about in terms of more basic, often concrete ideas. For example, he details the LOVE IS A JOURNEY metaphor, which enables circumstances and experiences concerning love to be thought of in terms of journeys. In contrast to the view that metaphors in dreams can elicit personal insight, Graveline and Wamsley (2015) state that there is “no evidence that dream content is any more symbolic than our waking cognition,” and that waking cognition and dream content are both a “relatively transparent amalgam of our daily thoughts, feelings, and experiences.” Contrary to this, we would hold that metaphors in waking life cognition and in dreams can often require considerable reflection to identify and understand, even sometimes needing the assistance of others.

### Dreaming and Empathy

As a result of the work on dreaming and personal insight, and so as to give individuals greater time to discuss and consider their dreams, Mark Blagrove and artist Julia Lockheart started an arts science collaboration, DreamsID (Dreams – Interpreted and Drawn; DreamsID.com), in which dreams are shared by the dreamer and discussed with him or her in a one hour consultation period, using the Ullman (1996) method, and simultaneously drawn and painted. After this session the dreamer is given the artwork capturing the dream, to display and return to across time, on their own or with significant others. The aim was to aid the socialization and consideration of the dream narrative and its metaphors across a time period of many months and even years. However, whereas the project was devised so as to elicit insights for the dreamer from the discussion and artwork, in undertaking the project it became apparent that there was an emotional effect on the discusser and artist, and on the significant others of each dreamer, who listen to and engage with the dream and artwork.

Previous researchers have investigated the effects of sharing dreams. In a sample of undergraduates, Vann and Alperstein (2010) found 97.9% had told a dream to someone else at least once, and that dreams were told in order to entertain, or to elicit a reaction, or to share, and concluded that dream sharing may serve as a means to bring individuals closer together. Ijams and Miller (2000) explored the reasons individuals offer for revealing dreams to an intimate other, and for concealing dreams. Results indicated that dream-disclosure enhanced feelings of intimacy and trust within established relationships, provided the others’ response was anticipated to be supportive and non-judgmental. Dream sharing can also enhance marital relationships through providing a forum for self-awareness and self-disclosure (Duffey et al., 2004).

Schredl and Schawinski (2010) found that about 14.5% of dreams are shared, mainly with romantic partners, friends, and relatives, and that the sharing is often associated with enhancement of relational intimacy and stress relief (for example, in the case of nightmares). Emotional intensity of the dream is the main predictor of social sharing for both negative and positive dreams (Curci and Rimé, 2008). Schredl et al. (2015a) found that, in a sample most of whom were psychology students, dreams were shared on average about 2 to 3 times per month, and the sharing was mostly with friends and spouses. At the time of completing questionnaires for the study about two thirds of participants had in the previous month told a dream to someone else, and two thirds had listened to a dream of someone else; furthermore, in the previous week, one third of participants had told a dream to someone else, and one third had listened to someone else’s dream. Regarding the last situation in which the participant had told one of his/her own dreams to another person, or listened to a dream told by another person, the three main motives for dream telling were “dream topic relevant for the interaction between the dreamer and the listener,” “extraordinary dream,” and “wish to understand the dream better.” The authors concluded that dream sharing is common and can affect the relationship between the dreamer and the recipient, and that reactions to dream accounts are more positively than negatively toned. Relevant here, although not addressing the sharing of dreams, are the findings of Selterman et al. (2014), that frequency with which participants dreamt of their romantic partners was positively associated with the extent to which they interacted with their partners, and that participants felt more love/closeness on days subsequent to dreaming about them, although dreams of infidelity resulted in less intimacy on subsequent days.

McNamara (1996) reviews evidence that REM sleep is designed to promote social bonding, that it may reactivate the systems utilized by infants to attach to a care-giver, and proposes that this may be reflected in dream content showing “bonding themes,” especially in individuals not currently attached. McNamara et al. (2001) found that insecurely attached participants were more likely to (a) report a dream, (b) dream “frequently,” and (c) evidence more intense images, with, they conclude, REM sleep and/or dreaming functioning, in part, to promote attachment. They propose that dreaming might shape daytime behaviors through activation and processing of persistent attachment related themes in dream content, given that relationship themes are quite frequent in dreams, and with the dream doing “serious emotional work,” often with unpleasant content.

From the above review it is clear that the sharing of dreams is common, and that positive interpersonal effects occur as a result of such sharing. It is thus plausible that dream sharing could elicit or be associated with empathy as part of these interpersonal effects. The most obvious reason why dreams would be able to have this relationship with empathy is their high social content. In the Social Simulation Theory (SST) of Revonsuo et al. (2016), dreams are a simulation of waking social life, with a mixture of characters, motivations, scenarios, and positive and negative emotions. Social interactions in dreams simulate the social skills, bonds, interactions and networks that we engage in during our waking lives. For example, Tuominen et al. (2019) found that at least one social situation was present in 83.5% of dream reports, and dreams were found to have more social content than corresponding waking life reports (63.8%). Domhoff and Schneider (2018) similarly characterize dream content as the embodied enactment of waking life conceptualizations and concerns, and report that only 6.5% of dream reports are not social simulations. Theirs is, however, a non-functional view, as they note the presence of long-term concerns in dreams, and social interactions with deceased loved ones across years and decades, and past misfortunes, which they say are not characteristic of SST “forward-looking social rehearsal.”

### Dreaming, Fiction, and Empathy

Aside from the considerable evidence that dream content is related to waking social life, a further component supporting a link between dreaming and empathy is that the dream acts as a piece of fiction, which is explored by the dreamer and others as part of the sharing process, and that, like literary fiction (Oatley, 1999; Matthijs Bal and Veltkamp, 2013), can induce empathy about the life circumstances of the dreamer. In the Mind in the Eyes test (Baron-Cohen et al., 2001), participants view 36 photographs, each showing only a person’s eyes, and choose from four adjectives to indicate what each photographed person was thinking and feeling. This is a behavioral test of empathy. Mar et al. (2006) used the Mind in the Eyes test to show a correlation between amount of reading fiction and trait empathy. This association was replicated by Mar et al. (2009), who showed that it was not due to personality variables related to both frequency of fiction reading and empathy. Matthijs Bal and Veltkamp (2013) showed that empathy was increased over a period of 1 week for people who read a fictional story, in comparison to a non-fictional piece, but that this effect only occurred if the reader was fully immersed into the story, “transported into this narrative world.” They state that the emotional response is greater than with non-fiction, because of the involvement with the characters and story, and because “the focus of fiction is primarily on eliciting emotions, rather than on presenting factual information…,” and that the reader sympathizes with the characters in the story, through taking the perspective of the characters, and experiences the events as if they are the reader’s own experience.

Our drawing a comparison between dreams and literary narrative does raise two questions, on the measurement of the narrative structure of dreams, and on the difficulties associated with deciding what is literary about literary narratives. On the first of these questions, Nielsen et al. (2001) quantified narrative progression in REM and NREM dreams using a story grammar tool to parse dream reports into their constituent components (actions, scenes, and characters) and to identify the causal precursors and consequences of the constituent actions. The two types of sleep did not differ with respect to the mere presence of story components. Episodic progression, that is, the minimal story unit, was defined as the occurrence of at least one character action for which both an initiating event and a consequence were also identified. A greater proportion of REM than NREM stage 2 reports contained at least one episodic progression, proportions were, respectively, 0.66 and 0.43. This significant difference was accounted for by the proportion of dreams with episodic progression being much higher (0.79) for late REM dreams of frequent dream recallers.

On the question of what is a literary narrative, Mar and Oatley (2008) include in this category novels, films, TV dramas, and theater, and state that these narratives model and abstract the human social world, and with the viewer, listener or reader undergoing a simulation of events. For a definition of literary narrative, they state that this includes “a series of causally linked events that unfold over time,” with “relationships among individuals and the navigation of conflicting desires.” They state that these narratives are “carefully crafted, written, and rewritten by authors intending their products for public consumption,” and “offering a form of cognitive simulation of the social world with absorbing emotional consequences for the reader.” Some of these characteristics of literary narrative obviously do not hold for dreams, but for the present paper the crucial characteristics that they have in common are that literary narratives and dreams are simulations of the waking social world, and that both can elicit engagement and emotion when told.

The brain basis for story production in dreams is detailed in Pace-Schott’s (2013) *Dreaming as a story-telling instinct*. The similarity between dreams and fictional stories is explored by States (1993), with dreams doing “much the same thing as the fiction writer who makes models of the world that carry the imprint and structure of our deepest concerns. And it does this by using real people, or scraps of real people, as the instruments of hypothetical acts.” States proceeds to describe “such narratives contributing to our formulation and recognition of patterns of experience,” and including scriptural violations or scripts in conflict. He compares dreams to two types of narrative, life itself, from which the dream borrows its content, and fiction, which is “waking dreams designed for other people,” and he cites Calvin Hall’s conclusion that people incorporated into dreams are those to whom we have mixed feelings, or some tension.

In their paper *The function of fiction is the abstraction and simulation of social experience*, Mar and Oatley (2008) state that “Engaging in the simulative experiences of fiction literature can facilitate the understanding of others who are different from ourselves and can augment our capacity for empathy and social inference.” They conclude that “In much of literature, the author challenges readers to empathize with individuals who differ drastically from the self,” and they propose that narrative fiction represents “learning through experience.” We emphasize that the functional SST and non-functional Domhoff views of dreaming both see the dream as fiction. Dreams are fictional because they have events that only very rarely copy waking life episodes (Fosse et al., 2003). Furthermore, in Vallat et al. (2017), an unknown dream environment occurs in just over 40% of dreams, and is significantly more frequent than an environment that is wholly or partly taken from waking life. (In contrast, other characters in the dream are more likely to be known than to be unknown or mixed).

### Testing the Empathy Theory of Dreaming

To date, no study has addressed the relationship between empathy and dream sharing, and between empathy and attitude toward dreams, although previous work has shown that dream recall frequency is correlated with empathy (Rabinowitz and Heinhorn, 1985), and attitude toward dreams is associated with the frequency of dream sharing (Schredl and Schawinski, 2010). We propose that dream sharing can elicit empathy toward the dreamer in the individuals with whom the dream is shared and discussed, and might increase empathy from the dreamer toward those with whom the dream is shared. The present Study 1 tests three hypotheses that follow from this proposed empathic effect of dream sharing: that trait empathy will be correlated with dream telling frequency, with frequency of listening to others’ dreams, and with positive attitude toward dreams.

Although a relationship between sharing dreams and empathy is plausible, there would be different possible explanations for this proposed relationship. Firstly, it may be that individuals high in empathy show an interest in the dreams of others, and due to connectedness to others wish to share their own dreams, and see dreams in general as worthwhile for sharing and considering. It may also be that there is an individual difference that is correlated with empathy and with these dream variables, such as, for example, Hartmann’s trait of thin boundariness (Hartmann et al., 1991). A third possible mechanism is that the sharing of dreams increases empathy and mutual understanding. From the literature on frequency and effects of dream sharing this is plausible, and especially as reactions to dream accounts are more positively than negatively toned (Schredl et al., 2015a). To address this we conducted Study 2, the aim of which was to assess changes in empathy following dream discussion, differentiating between empathy by a dream sharer toward their discusser, and empathy by the discusser toward the dream sharer. The primary hypothesis is that the discusser will have increased empathy toward the dream sharer. A second hypothesis is that the dream sharer will have increased empathy toward the discusser. It is unclear whether the two members of the dyad will differ in their change in empathy due to the discussion, and so there is no hypothesized difference between them in this regard.

## Study 1

### Methods

#### Participants

A total of 160 participants (120 females, 40 males; mean age = 21.30 years, *SD* = 4.70) were recruited from social media sites and from the Swansea University Department of Psychology’s experiment participation scheme. All participants gave written informed consent in accordance with the Declaration of Helsinki. The protocol was approved by the Research Ethics Committee, Department of Psychology, Swansea University. The study was described to recruits as being about “reading, emotions and dreaming.” We included questions about reading habits in the study so that it would be unclear to participants what our hypotheses were.

#### Procedure and Materials

Participants completed online the Toronto Empathy Questionnaire (TEQ; Spreng et al., 2009) and the Mannheim Dream Questionnaire (MADRE; Schredl et al., 2014). The TEQ has 16 items, each scored on a 5 point scale, anchored at Never (0) and Always (4), with half the items negatively scored. Example items are:

“It upsets me to see someone being treated disrespectfully.”

“I become irritated when someone cries.”

The total score is the sum of all item scores and can vary between 0 and 64.

The items used from the MADRE were:

“How often have you recalled your dreams recently (in the past several months)?” Participants responded on a 7 point scale, using points ranging from “almost every morning” (7) to “never” (1). The other points on the scale are: 6 = Several times a week; 5 = About once a week; 4 = Two or three times a month; 3 = About once a month; 2 = Less than once a month.

“How often do you tell your dreams to others?” Participants responded on an 8 point scale, using points ranging from “several times per week” (8) to “never” (1). The other points on the scale are: 7 = About once a week; 6 = Two to three times a month; 5 = About once a month; 4 = About two to four times a year; 3 = About once a year; 2 = Less than once a year.

The MADRE assesses Attitude toward Dreams (ATD) with 8 items each scored on a 5 point scale from “Not at all” (0) to “Totally” (4). The items are:

“How much meaning do you attribute to your dreams?”

“How strong is your interest in dreams?”

“I think that dreams are meaningful.”

“I want to know more about dreams.”

“If somebody can recall and interpret his/her dreams, his/her life will be enriched.”

“I think that dreaming is in general a very interesting phenomenon.”

“A person who reflects on her/his dreams is certainly able to learn more about her/himself.”

“Do you have the impression that dreams provide impulses or pointers for your waking life?”

The scale ranges from 0 to 32.

An item not present on the MADRE was added:

“How often do you listen to others telling their dreams to you?” Participants responded on the 8 point scale, with points ranging from “several times per week” (8) to “never” (1).

#### Analyses

Pearson partial correlations were conducted between the trait empathy and dream variables, with age and sex partialled out. Analyses using a median split for the empathy variable were then conducted, with difference on dream variables computed by ANOVA for the high/low empathy categories, and with η^2^ calculated as effect size.

### Results

Table 1 shows descriptive statistics of the trait empathy and dream variables. These variables had a normal distribution with skewness < 0.92.

**TABLE 1:** Descriptive statistics of trait empathy and dream variables.

	**Mean**	***SD***	**Min**	**Max**
Trait Empathy	48.13	7.93	15	64
Attitude toward dreams	20.33	6.34	0	32
Frequency of telling dreams^1^	5.53	1.89	1	8
Frequency of listening to dreams^1^	5.61	1.74	1	8
Dream recall frequency^2^	5.04	1.33	1	7

*${}^{1}$8 point scale, using points ranging from “several times per week” (8) to “never” (1). ${}^{2}$7 point scale, using points ranging from “almost every morning” (7) to “never” (1).*

Table 2 shows sex differences for the empathy and dream variables, with independent samples *t*-test statistics for the differences. Females scored significantly higher on all variables, except for marginally higher for frequency of listening to dreams of others.

**TABLE 2:** Sex differences for trait empathy and dream variables, with independent samples *t*-test statistics for the differences.

	**Male**		**Female**			
	**Mean**	***SD***	**Mean**	***SD***	***t*(158)**	***p***
Trait Empathy	44.93	8.81	49.20	7.35	3.028	0.003
Attitude toward dreams	17.78	6.38	21.18	6.11	3.012	0.003
Frequency of telling dreams^1^	4.75	2.11	5.79	1.75	3.094	0.002
Frequency of listening to dreams^1^	5.18	2.01	5.76	1.62	1.853	0.066
Dream recall frequency^2^	4.68	1.29	5.16	1.33	2.007	0.046

*${}^{1}$8 point scale, using points ranging from “several times per week” (8) to “never” (1). ${}^{2}$7 point scale, using points ranging from “almost every morning” (7) to “never” (1).*

We next tested the associations between trait empathy and the dream variables. As reported above, males and females differed significantly on empathy, ATD, and dream telling frequency, with dream listening frequency marginally greater for females. We therefore partialled out sex from the correlations. For empathic concern and perspective taking, there is an inverse-U-shaped pattern across age, with middle-aged adults reporting higher empathy than both young adults and older adults (O’Brien et al., 2012). Our sample was aged 18 – 48 years, and did show this expected positive relationship between age and empathy within this age range (*r* = 0.13, *p* < 0.05 one-tailed). We thus also partialled out age from the correlations. The Pearson partial correlations are presented in Table 3, and confirm the hypothesized associations of trait empathy with ATD and with frequencies of telling and listening to dreams.

**TABLE 3:** Pearson partial correlation co-efficients between trait empathy and dream variables, with age and sex partialled out, dfs = 156.

	**Empathy**	**ATD**	**Fr Tell**	**Fr List**
Attitude toward dreams	$0.29^{*,**}$			
Frequency of telling dreams	$0.32^{*,**}$	$0.26^{*,**}$		
Frequency of listening to dreams	0.14^*^	0.20^∗∗^	$0.57^{*,**}$	
Dream recall frequency	0.19^*^	$0.46^{*,**}$	$0.46^{*,**}$	$0.31^{*,**}$

^*^p < 0.05, ^∗∗^*p* < 0.01, and ^∗∗∗^*p* < 0.001 (ps are one-tailed).

As dream recall frequency was significantly associated with dream telling frequency, trait empathy and attitude toward dreams, the correlations of trait empathy with frequency of telling dreams to others and with positive attitude to dreams could thus be confounded by frequency of the dreamer recalling dreams. In a further analysis dream recall frequency was thus also partialled out; the correlations of trait empathy with frequency of dream telling (*r* = 0.26, *p* < 0.001, *df* = 155) and with ATD (*r* = 0.24, *p* < 0.005, *df* = 155) remained significant.

In order to investigate whether ATD accounts for further variance in empathy, beyond that explained by the two dream sharing variables, and whether the dream sharing variables account for empathy variance beyond that explained by ATD, further partial correlations were conducted, with these variables partialled out. Table 4 shows that ATD remains significantly associated with empathy when dream sharing variables are partialled out, and frequency of telling dreams, but not listening to dreams, remains significantly associated with empathy when ATD is partialled out.

**TABLE 4:** Pearson partial correlation co-efficients between trait empathy and dream variables, with age, sex, dream sharing and ATD variables partialled out, dfs = 155.

	**Empathy**	**Variables partialled out**
Attitude toward dreams	0.23^∗∗^	Dream telling frequency, age, sex
Attitude toward dreams	$0.27^{*,**}$	Dream listening frequency, age, sex
Frequency of telling dreams	0.26^∗∗^	ATD, age, sex
Frequency of listening to dreams	0.08	ATD, age, sex

*^∗∗^*p* < 0.01 and ^∗∗∗^*p* < 0.001 (ps are one-tailed).*

Analyses using a median split for the empathy variable were then conducted. Median value for empathy = 49.0, nine participants fell on the median and were excluded. Means (SDs) of empathy for the below (*n* = 77) and above (*n* = 74) median groups were 41.97 (6.46) and 54.43 (3.63) respectively. The below and above median groups were compared for ATD and dream sharing variables using ANOVA, with age and sex partialled out. Table 5 shows that the below and above median groups differ significantly on ATD and dream telling frequency, and marginally on dream listening frequency.

**TABLE 5:** Differences between below (*n* = 77) and above (*n* = 74) median trait empathy groups on the ATD, dream sharing and dream recall variables, with ANOVA statistics and η^2^ effect size for each dream variable.

	**Below median empathy**		**Above median empathy**		**Inferential statistics for comparison of below and above median groups**		
	**Mean**	***SD***	**Mean**	***SD***	***F*(1,146)**	***p***	**$\eta^{2}$**
Attitude toward dreams	18.82	7.01	21.80	4.99	7.076	0.009	0.046
Frequency of telling dreams	4.99	2.09	6.08	1.59	9.654	0.002	0.062
Frequency of listening to dreams	5.42	1.86	5.82	1.63	3.790	0.053	0.025
Frequency of recalling dreams	4.84	1.37	5.24	1.30	3.745	0.055	0.025

To address the role of gender in the inferential findings we included the main effect of gender and the interaction of gender with empathy in the ANOVAs. There is a main effect of sex on frequency of telling dreams (η^2^ = 0.035, *p* = 0.023) and on ATD (η^2^ = 0.061, *p* = 0.002) and no main effect of sex on frequency of listening to dreams (η^2^ = 0.013, *p* = 0.174) nor frequency of dream recall (η^2^ = 0.012, *p* = 0.179). There was no interaction of sex with empathy as a predictor of these dream variables (listening, η^2^ = 0.017; telling, η^2^ = 0.003; ATD, η^2^ = 0.003; dream recall frequency, η^2^ = 0.013, all ps > 0.1). Females thus score higher on the empathy and dream variables than do males but there is no significant difference between males and females in their relationships between empathy and the dream variables.

## Study 2

### Methods

#### Participants

27 pairs of participants were recruited to the study, each pair had applied together, as friends or in a relationship, knowing that one would be sharing dreams and the other of the pair would discuss the dreams with them. The sharer was identified as the member of the pair with highest retrospective dream recall frequency. There was data loss for one dream sharer, and thus 53 participants overall were included in the analyses (22 males and 31 females; 18 – 23 years, mean age = 20.97 years, *SD* = 1.35), comprising 26 dream sharers and 27 discussers. All participants gave written informed consent in accordance with the Declaration of Helsinki. The protocol was approved by the Research Ethics Committee, Department of Psychology, Swansea University.

#### Procedure

At the start of the study each participant completed online an adapted version of the 12-item Shen (2010) state empathy scale (see below), regarding empathy toward the other member of the pair. This produced a baseline empathy score for each participant toward the other member of their pair. Upon having a dream, the dream sharer arranged to meet the other member of the pair so as to discuss the dream with them. The discussion followed the stages of the Ullman (1996) dream appreciation technique, written instructions for which were given to dream sharers and discussers at the start of the study. The stages of the technique are as follows:

(1) Reading of the dream aloud by the dreamer, and clarification of the dream report by the discusser asking questions of the dream sharer.
(2) Brief statement by the discusser of what feelings they would have experienced if the dream were their own, and of how the discusser would see the dream in terms of their own life.
(3) The dream sharer can respond to anything said in stage 2, and then describes his/her waking life as a context for the dream, with particular emphasis on recent experiences and concerns.
(4) The discusser reads back the dream to the dreamer, in the second person, so that any additional information about the dream or the dreamer’s waking life can be obtained.
(5) Orchestration, in which the dream sharer and discusser suggest connections between information that the dreamer has given about his or her dream and information the dreamer has given about the dreamer’s life.

The discussion duration was set at 15–30 min. Each participant completed the state empathy scale after the dream discussion. During the study the majority of participants had one dream discussion. (Some participants, progressively fewer each time, had 2 to 5 dream discussions as part of an unsuccessful attempt by the experimenters to obtain sufficient data for repeated-measures analysis.) The empathy score following the last or only dream discussion is used as the post-intervention measure, and compared to the empathy score measured at baseline.

#### Materials

Adapted Shen (2010) state empathy scale.

Each item is scored on a 0 – 10 scale, where 0 = not at all and 10 = completely.

(1) My friend’s/partner’s emotions are genuine.
(2) I experience the same emotions as my friend/partner.
(3) I have a similar mood to my friend/partner.
(4) I can feel my friend’s/partner’s emotions.
(5) I can see my friend’s/partner’s point of view.
(6) I recognize my friend’s/partner’s situation.
(7) I can understand what my friend/partner goes through.
(8) My friend’s/partner’s reactions are understandable.
(9) When I talk to my friend/partner, I am fully absorbed.
(10) I can relate to what my friend/partner goes through.
(11) I can identify with the situations my friend/partner describes to me.
(12) I can identify with my friend/partner.

Scores on the scale range from 0 to 120.

#### Analyses

It was predicted that each member of the pair will have a significant increase in empathy toward the other, and so these changes are assessed by one-tailed paired samples *t*-test. A difference between the change score of sharer and change score of discusser is not hypothesized, and so is assessed by two-tailed independent samples *t*-test. Effect size where a paired-sample *t*-test achieves significance (*p* < 0.05) was calculated as d_z_ = t/sqrt (n) (Lakens, 2013) where n = number of participants. Following Cohen (1988, p. 40 and p. 46), thresholds for d_z_ are small effect = 0.14, medium effect = 0.35, and large effect = 0.57. Effect size for the independent sample *t*-test was calculated as d_s_ = t × sqrt (1/n_1_ + 1/n_2_), where n is the sample size for each independent group, and for which Cohen (1988) gives thresholds of small effect = 0.2, medium effect = 0.5, and large effect = 0.8.

### Results

Table 6 shows that, as hypothesized, the dream discusser had an increase in empathy from baseline toward the dream sharer as a result of the discussion, with medium effect size of d_z_ = 0.343. The dream sharer had a non-significant decrease in empathy toward the discusser. The discusser had a significantly greater change in empathy score compared to the dream sharer, with medium effect size of d_s_ = 0.554.

**TABLE 6:** Baseline and post-dream discussion empathy of dream sharer (*n* = 26) and dream discusser (*n* = 27) toward each other, and change in empathy from baseline for sharer and discusser.

	**Baseline empathy**		**Post-dream discussion empathy**		**Paired samples *t*-test**		**Change in empathy from baseline**		**Independent samples *t*-test**
**Role**	***M***	***SD***	***M***	***SD***	***t***	***p*(1-tail)**	***M***	***SD***	
Sharer	85.54	12.18	83.38	9.17	*t*(25) = 1.037	0.310	−2.15	10.59	*t*(51) = 2.017 *p* = 0.049 (2-tail)
Discusser	78.67	17.20	82.93	15.99	*t*(26) = 1.780	0.044	+4.26	12.44	

*Number of sharer/discusser pairs = 27, data were lost for one dream sharer. Baseline empathy of sharers and discussers did not differ significantly [*t*(51) = 1.672, *p* = 0.10].*

## Discussion

The three hypotheses of Study 1 were confirmed: trait empathy was found to be significantly associated with frequency of listening to the dreams of others, frequency of telling one’s own dreams to others, and positive attitude toward dreams. However, when ATD and dream sharing variables were used as covariates in the respective correlations the findings were that it was dream telling frequency and ATD that remained significantly associated with empathy. These relationships may be solely correlational, with high empathy people choosing to share dreams, or due to some other personality measure such as thin boundariness (Hartmann et al., 1991) being associated with empathy and with dream variables. The relationship between dream sharing and empathy also involves a belief in dreams being a worthwhile subject of deliberation and discussion, rather than solely a simple frequency of engaging in dream sharing. The possibility of a causal relationship was addressed in Study 2, where, as hypothesized, the dream discusser had a significant increase in empathy toward the dream sharer as a result of discussing dreams following the Ullman technique. However, contrary to our expectations, the dream sharer had a small decrease in empathy toward the discusser, and indeed the discusser had a significantly greater change in empathy as a result of the discussion than did the sharer. These latter findings, in retrospect, can be understood in that the dream sharer is addressing their own dream and own life during the discussion process, and so would not necessarily have a significant change in empathy toward the discusser. An increase in empathy for both members of a pair would thus need them to take turns in sharing and discussing.

The results from the two studies thus provide support for the empathy theory of dreaming. However, although accepting the results, a sceptical view could quote the often repeated claim that “There is nothing more boring than listening to someone else’s dream!” The sceptical view would state that, although on an everyday basis (as opposed to formal dream sharing groups) people may choose to share their dreams with others, it could be argued that this is done so as to tell a particularly strange or unusual dream, and that while the other person will listen, they rarely engage with the dream beyond their mere (and at times polite) listening. Indeed, from the Study 1 data and review here, at best most people will share their dreams about once or twice a month – and this amongst people likely favorable toward dreams in general. Furthermore, people may agree to listen to others wanting to tell them a dream, as doing otherwise would be exceedingly rude. It could be argued that while positive interpersonal effects can occur, this might not be so for a majority (or even sizable minority) of spontaneous or everyday dream sharing. Furthermore, aside from maybe lacking genuine interest in the dreams that we are told, most people are not versed in ways of constructively reacting to and engaging with the relayed dream. If this is indeed the case, it may be argued that most people will do little more than listen to the dream – and that this, in fact, may well be the normal response to most shared dreams in everyday settings.

In response to this sceptical view, it may well indeed be that only a minority of recalled dreams are shared (approximately 14% of all recalled dreams were shared in a study by Schredl and Schawinski, 2010). Yet, it may be possible that even the small number of shared dreams bears an effect on empathy. In previous studies, 35% of respondents representing the general population share dreams at least monthly, and about 10% weekly or several times per week (Schredl et al., 2014), and in children and adolescents, sharing seems to be more frequent (Georgi et al., 2012). Thus, a significant proportion of the population engages in dream sharing regularly. Whereas Schredl et al. (2015a) found that negatively toned dreams are shared more often than positively toned dreams, for the most recent time a participant had heard someone else’s dream, joy was a response for 63% of occasions. The main motives for sharing dreams include the dream topic being relevant for the interaction between dreamer and listener, the dream being extraordinary, for entertainment, to inform others of what is going on in the sharer’s mind, to understand what the dream means, and interest in the opinion of others (Olsen et al., 2013). In cases of nightmares and negative dreams, dream telling is a means to seek relief for negative emotions elicited by the nightmare (Schredl and Göritz, 2014). Sharing strange and unusual dreams, or emotionally highly salient dreams, may be thus more frequent than sharing mundane dreams, but this probably also applies to sharing waking events as well. That the gender difference in dream sharing is related to frequency of sharing emotional experiences and to sex role orientation (femininity/expressivity), rather than dream-related variables such as dream recall frequency and ATD (Schredl et al., 2015b), suggests that dream sharing can be a positive self-disclosure process.

Furthermore, data on how listeners respond to dream sharing show that boredom is not the most common reaction. Schredl et al. (2015a) report that laughter/amusement and sympathy are the most common reactions, accounting for about 35% and 23% of responses respectively, with only 10% of reactions being neutral or there being no response, and with the most common emotions associated with dream listening being astonishment, joy, grief, and the dream being seen as strange. In a recent study, Schredl and Göritz (2018) found that 21% of respondents report enjoying listening to dreams “very much,” and 35% “much,” with 6% responding with “not at all.” Regarding the last-remembered situation in which a dream was told to the participant, the emotional reaction was most often positive (49.2%), with 45% of the participants rating the last listening experience as neutral, and only 2.1% as negative. Although this online sample may have been biased toward people who are interested in dreams, the sample was representative of the general population and with heterogenic demographic backgrounds (sample was of 935 women and 655 men, mean age = 51.20, range 17 to 93 years). It is thus plausible that listeners are not uninterested in other people’s dreams, nor emotionally indifferent. This is also supported by Schredl and Göritz’s (2018) findings that after the most recent dream listening situation percentages of further responses were, “I thought about the dream” (19%), “I talked again with the person about the dream” (20%), “I feel more close to the person who shared the dream” (13%), “I talked with others about the dream” (5%), and “I learned something about myself” (1.6%), these being overall marginally more frequent reactions than “I did not do anything more with the dream” after listening to it (45%). In summary, the findings reviewed here support a view that in general listening to the dreams of other people is a positive experience emotionally and in terms of social interaction.

### Dreaming and Narrative

Using a microstructural approach, Montangero and Cavallero (2015) found that dreams are predominantly “continuous,” but with most dreams having one or more “complications,” defined as unexpected events creating change and a certain tension. They state that continuity and complications are both necessary for narrative, but that dream reports were not found to be structured like canonical stories, which would have semantic unity, growing tension, and the presence of an ending. Similarly, Montangero (2012) reviews theories and evidence on dreaming and narrative to show that although comprising sequences of usual and unusual events, with protagonists and motivations, dreams do not in general show the well organized structure of canonical stories. For the current paper, it may be that this lesser level of narrative is sufficient for the empathic effects of dream sharing. It may even be, as noted by Montangero (2012), that although the lack of executive functioning in sleep results in dreams not being formally story-like, it allows creative possibilities for representing our concerns in dreams. The manner of representation in dreams is further addressed by Walsh (2010), who describes the ambiguous status of dreaming, as experience and as narrative, and where there is a reciprocal process of creation and reception. This experiential component of dreaming makes it a special case of narrative, and one for which he suggests theoretical accounts of narrative may have to accommodate.

Relevant also to a loose form of narrative is Bulkeley’s (2019) proposal that dreaming is imaginative play in sleep. He reviews play across species, and shows that dreaming shares the behavioral components of waking life human play, including practice, rehearsal, opening the mind to new possibilities beyond ordinary experience, and incorporating issues and concerns from waking social life. Bulkeley suggests that dreaming has many of the same functions as waking life play, and that dreaming may thus have been selected for this during human and mammalian evolution. The current paper, however, proposes a further use for dreams, that, when shared, they have effects on others, and which go beyond, albeit building on, the playful stretching of the mind of the dreamer that Bulkeley describes.

### Evolution and Dream Sharing

There have been many theories that propose a within-sleep function of dreaming, for example, of threat simulation (Revonsuo, 2000), social simulation (Revonsuo et al., 2016), fear extinction (Levin and Nielsen, 2007), and the creation of weak or novel memory associations (Hartmann, 2011; Wamsley and Stickgold, 2011). These all hold that at the point of dream production, dream function occurs. What is proposed by the empathy theory is that there is (also) an effect that occurs later than dream production, when we are awake and share the dream. The empathy theory proposes that the often emotional dream simulation, if recalled, may have a lasting effect on the dreamer after sleep (through self-reflection), but also on significant others who are told and engage with the dream.

Possibly only McNamara et al.’s (2007) Costly Signaling Theory (CST) of dream recall and sharing has similarly proposed social effects of dream sharing on others from an experimental psychology standpoint, although we appreciate that this has been a frequent view in anthropology (e.g., Tedlock, 1987). The CST states that only signals or communications that are costly to produce will be seen as believable and not faked, and that, just as antlers are costly, so is REM sleep. Dreams are seen to give true information about the dreamer because they are involuntary and emotional, the dreamer signals on sharing that the dream was not invented, and shows that he or she can overcome the emotions of the dream. Dream sharing is thus a sign of strength or of good genes. As with the empathy theory, the CST posits that the long term result of advertising this honesty in communication is better social interactions for the individual. However, by contrast, the empathy theory emphasizes that others will come to an appreciation of the life circumstances and even vulnerabilities of the dreamer, rather than be impressed by their strength. Other differences between the empathy theory and CST are that the former prioritizes fiction and narrative that has to be explored by the dreamer and recipients, and that it is accepting of the fact that dreams are fakeable, in that a fake dream can still fulfil the self-disclosure function. In the empathy theory there are thus similarities between the told dream simulation and blushing, in that both signal the emotional state of the dreamer/blusher to others. Because the blush is involuntary, it is a believable signal about regret and about not wishing to transgress in the future, amongst the signaling of other emotions.

So far the proposal for this empathy effect of dream sharing, with mediation by the fictional content of dreams, is plausible and accords with the correlational and experimental findings reported here, but we now turn to a more speculative aspect. We speculate that the fictional/story-like characteristic of dreams may have been utilized or even enhanced in human evolution, and in human sexual selection, as part of the selection for emotional intelligence and empathy. This would be on a timescale similar to that for the evolution of language and storytelling as part of group cohesion and cooperation in humans (Smith et al., 2017).

A key component of the “mating mind” hypothesis (Miller, 2000) is that a wide range of behaviors that require significant intellectual ability, and are wasteful or irrelevant in terms of survival – behaviors such as music, complex language, or art - are actually honest signals of intelligence that have been selected for over a relatively short period of human history. Indeed, cranium size of human fossils show a steady and consistent growth over a period of around three million years, until anatomically modern humans emerged some 300,000 years ago with an approximately tripled brain size (Du et al., 2018). Arguably, this is too short a time for natural selection to increase brain size due to environmental pressure, and indeed, a larger cranium carries significant mortality costs during childbirth (Rosenberg, 1992). As such, sexual selection is likely to be the mechanism that has driven the increased brain size required for these behaviors.

A number of complex social behaviors seem to increase the attractiveness or likeability of the producer by those witnessing the behavior. For example, the creativity of male story-tellers contributes to their attractiveness beyond physical appearance (Watkins, 2017), and they are seen as more appealing when completing verbal and physical tasks (Prokosch et al., 2009). Related, these traits show significant genetic correlations, indicating heritability – a key component of any evolutionarily relevant trait (Verweij et al., 2014). More specifically, individuals skilled at story-telling, a behavior clearly associated with creativity, have greater mating success and are preferred social partners for cooperation, extending the benefits outside the domain of sexual reproduction (Smith et al., 2017). As such, creative individuals may benefit from creative signals both directly, in that it increases attractiveness, or indirectly, in that increased cooperation leads to greater networks.

Dreams and dream-sharing might have contributed to story-telling abilities (such as by providing material for stories) and possibly to empathy eliciting behaviors, with selection to increase positive social exchanges, mating related or otherwise, such as described in Dunbar’s (2016) social brain hypothesis. This hypothesis holds that complex social life in primates has been the driving force behind increasing brain size, and that the relationship between brain size and group size is mediated, in humans at least, by mentalizing skills, which includes mentalizing about others and empathy. Dreams themselves might have originated from memory consolidation or threat rehearsal or other functions, or indeed might be no more than a spandrel, an epiphenomenon of sleep (Flanagan, 2000). However, as described by Barrett (2007), spandrels can become useful, in that: “A useful ‘spandrel’ immediately begins to evolve. To the extent that REM sleep supports dreaming, it emerged long ago – about the same time mammals appeared. There’s been plenty of time for refinement – including of resulting cognitions.” Thus, dreams may have been co-opted to add to story-telling abilities and empathy in humans. This possible empathy and bonding function of dreams would utilize the social characteristics of the simulation/dream, when the simulation/dream, on waking, is told to others. It may also utilize the architecture of sleep, in that the later REM periods, and especially the period closest to waking, are the longest, with greater story-like complexity and episodic progression in REM than in NREM dreams (Nielsen et al., 2001), and greater elaboration, length and complexity of dream-stories in later REM periods (Cipolli and Poli, 1992; Cipolli et al., 2015).

### Implications for Consciousness

We can extend the arguments about dreaming in the present paper to consciousness more generally. Oatley (2016) in *Fiction: Simulation of social worlds*, states that people who read fiction improve their understanding of others, because fiction has complex characters and circumstances that we might not encounter in daily life. He concludes: “While some everyday consciousness can remain inside the individual mind and be externalized in small pieces during conversations, fictional stories can be thought of as larger pieces of consciousness that can be externalized by authors in forms that can be passed to others so that these others can internalize them as wholes, and make them their own.” The present paper is proposing that dreams can, like fictional stories, be passed to others who internalize them as wholes. But what is being said of dreaming consciousness could also be said of the scenarios and narratives present in waking consciousness. A function of human consciousness could thus be that its content and narratives can be passed to and engaged with by others, resulting in second-person social benefits, and not just experienced in the first-person for access to (Block, 1995) and binding of emotional and cognitive processes.

### Limitations

For Study 1, we acknowledge that whereas we used a uni-dimensional empathy questionnaire future research on the empathy theory should use measures that differentiate cognitive and affective dimensions, such as the Empathy Quotient (Baron-Cohen and Wheelwright, 2004) and the Questionnaire for Cognitive and Affective Empathy (Reniers et al., 2011). We would hypothesize that dream sharing is associated with both these dimensions. We also acknowledge that in this first study on this issue of dream-sharing and empathy we have used two simple behavioral measures of dream-sharing, that is, frequency of telling dreams and frequency of listening to dreams. We thus did not address interactive factors, such as motives for sharing a dream, levels of being attuned to or skilled in the discussion of dreams or other personally meaningful texts, and characteristics of dreams that are shared (or not shared). For example, Curci and Rimé (2008) differentiate the disclosure of emotionally positive and negative dreams and address the emotional intensity of shared dreams. We also did not examine the nature of the relationship with the person with whom the dream sharing is occurring (e.g., partner, friend, work colleague), nor individual differences in self-disclosure, nor factors that might affect disclosure, such as attachment styles. For example, individuals with insecure attachment orientations have been shown to limit their use of emotional disclosure as a means of emotion regulation (Garrison et al., 2012). Some of these factors might result in higher correlations of dream-sharing with empathy. In future work it will be necessary to address all these factors so as to elucidate mechanisms behind the relationship between dream-sharing and empathy, to compare the empathy theory with other theories of dreaming, and to relate this theory and findings to the more general literature on self-disclosure.

For study 2, again, a limitation is that we used a uni-dimensional empathy questionnaire. The main limitation, however, is that we did not use a comparison condition in which some meaningful narrative material other than a dream report was used as the basis for the discussion. Comparison conditions in future work could be the discussion of a recent significant event in the life of the dreamer, as used in Edwards et al. (2015), or the dream sharer telling someone else’s dream to the listener in their dyad (Hill et al., 1993).

Regarding the general theory that dreaming is a form of fiction, we accept that we have not used or addressed sophisticated or differentiated theories of metaphor and of literary narrative, and have instead used simple versions of these concepts. Although we have referred to Lakoff and Johnson’s (1980) theory of conceptual metaphor, future work should have regard to and assess metaphors on such variables as conventionality and aptness (Thibodeau and Durgin, 2011) and surprisingness, comprehensibility, and metaphoricity (Thibodeau et al., 2016). We have also not differentiated metaphor from other literary tropes, such as irony and synecdoche, as described by States (1989, 1997), instead going no further than the highly simple conceptualisation that metaphors are a non-literal representation of waking life, and which occur, as stated by Antrobus (1977), when the dream changes the context or attributes of waking life experiences. We acknowledge that a more differentiated account of non-literal representations is needed, and especially as the different tropes might afford different levels of creative restructuring of prior experience as part of the memory consolidation and cognitive flexibility and recombination functions of sleep and/or dreaming (Wagner et al., 2004).

### Summary

Study 1 found that trait empathy is significantly correlated with frequency of telling dreams to others, frequency of listening to others’ dreams, and positive attitude toward dreaming, and Study 2 found that dream sharing increases empathy in the listener/discusser toward the dream sharer. We propose that the dream acts as a piece of fiction, which others can explore with the dreamer and that, like literary fiction, can then induce interest in and empathy about the life of the dreamer. Increased dream telling across society might decrease differences between countries in levels of empathy (Chopik et al., 2017) and counteract current societal decreases in empathic concern and perspective taking, the main two components of empathy (Konrath et al., 2011).

## Ethics Statement

The studies were carried out in accordance with the recommendations of the Research Ethics Committee, Department of Psychology, Swansea University with written informed consent from all subjects. All subjects gave written informed consent in accordance with the Declaration of Helsinki. The protocols were approved by the Research Ethics Committee, Department of Psychology, Swansea University.

## Author Contributions

MB, JL, MC, AJ, and KV conceived the manuscript. MB, JL, MC, and SH designed the Study 1. SH collected the data for Study 1. SH and MB analyzed the data for Study 1. MB, JL, and MC designed the Study 2. MB analyzed data for Study 2. MB, JL, MC, AJ, and KV wrote the manuscript.

## Conflict of Interest Statement

The authors declare that the research was conducted in the absence of any commercial or financial relationships that could be construed as a potential conflict of interest.
